# CXCL10 and neopterin in CSF are candidate prognostic biomarkers for HTLV-1-associated myelopathy/tropical spastic paraparesis

**DOI:** 10.1186/1742-4690-11-S1-P18

**Published:** 2014-01-07

**Authors:** Yoshihisa Yamano, Tomoo Sato, Hitoshi Ando, Natsumi Araya, Naoko Yagishita, Junji Yamauchi, Ariella Coler-Reilly, Atae Utsunomiya, Steven Jacobson, Shuji Izumo

**Affiliations:** 1 Institute of Medical Science, St. Marianna University School of Medicine, Kawasaki, Japan; 2 Department of Hematology, Imamura Bun-in Hospital, Kagoshima, Japan; 3 Viral Immunology Section, Neuroimmunology Branch, National Institutes of Health, Bethesda, MD, USA; 4 Molecular Pathology, Center for Chronic Viral Diseases, Kagoshima University, Kagoshima, Japan

## 

Human T-lymphotropic virus type 1 (HTLV-1) -associated myelopathy/tropical spastic paraparesis (HAM/TSP) is a chronic neuroinflammatory disease. Since the disease course of HAM/TSP varies among patients, there is a dire need for biomarkers capable of predicting the rate of disease progression for earlier detection of high-risk patients. However, there have been no studies to date that have compared the prognostic values of multiple potential biomarkers for HAM/TSP. Peripheral blood and cerebrospinal fluid (CSF) samples from HAM/TSP patients and HTLV-1-infected control subjects were obtained and tested for several potential biomarkers, including chemokines and other cytokines, and 8 optimal candidates were selected based on receiver operating characteristic (ROC) analysis. Next, we evaluated the relationship between these candidates and the rate of disease progression in HAM/TSP patients, beginning with a Training Set of 30 patients and proceeding to a Test Set of 23 patients. We defined “deteriorating HAM/TSP” as distinctly worsening function (≥ 3 grades on Osame’s Motor Disability Score (OMDS)) over 4 y and “stable HAM/TSP” as unchanged or only slightly worsened function (1 grade on OMDS) over 4 y, and we compared the levels of the candidate biomarkers in patients divided into these 2 groups. The CSF levels of chemokine (C-X-C motif) ligand 10 (CXCL10), neopterin and the CSF cell count were well-correlated with disease progression, better even than HTLV-1 proviral load in PBMCs. Importantly, these results were cross-validated using the Test Set. Therefore, the CSF levels of CXCL10 and neopterin represent the most viable candidates for HAM/TSP prognostic biomarkers.

